# Current state of interventional procedures to treat pernicious placenta previa accompanied by placenta accreta spectrum: A review

**DOI:** 10.1097/MD.0000000000034770

**Published:** 2023-09-15

**Authors:** Hu Zhao, Qiong Wang, Mou Han, Xue Xiao

**Affiliations:** a Department of Gynecology and Obstetrics, West China Second University Hospital, Sichuan University, Chengdu, China; b Department of Obstetrics and Gynecology, Chengdu Women and Children’s Central Hospital, Chengdu, China; c Department of Intervention, Chengdu Women and Children’s Central Hospital, Chengdu, China.

**Keywords:** internal iliac artery embolization, Interventional procedures, intra-abdominal aortic balloon occlusion, pernicious placenta previa, postpartum hemorrhage, prophylactic balloon occlusion of the internal iliac artery, uterine aortic embolization

## Abstract

Pernicious placenta previa (PPP) accompanied by placenta accreta spectrum (PAS) is a life-threatening placental implantation that causes a variety of complications, including antepartum hemorrhage, postpartum hemorrhage, hemorrhagic shock, preterm birth, and neonatal asphyxia. Along with continuous improvements in medical technology, interventional procedures have been widely used to prevent intraoperative hemorrhage associated with PPP. The commonly used interventional procedures include abdominal aorta clamping, prophylactic balloon occlusion of the internal or common iliac arteries, and uterine artery embolization. The above-mentioned interventional procedures have their respective advantages and disadvantages. The best procedure for different situations continues to be debated considering the complex pattern of blood supply to the uterus in patients with PPP. The specific choice of interventional procedure depends on the clinical situation of the patient with PPP. For grade III PAS, the need for uterine artery embolization is assessed based on blood loss and preoperative hemostatic effect following abdominal aorta clamping. Repair or hysterectomy may be performed following uterine artery embolization if there is a hybrid operating room for grade III PAS patients with extensive sub-serosal penetration of the uterus and repair difficulty. For grade II PAS (shallow placental implantation), prophylactic balloon occlusion may not be necessary before surgery. Uterine artery embolization can be performed in case of postoperative hemorrhage.

## 1. Introduction

Postpartum hemorrhage is a leading cause of parturient death, accounting for 49.2% of all parturient deaths.^[1]^ Because cesarean section rate continues to be high, pregnancy in a scarred uterus becomes increasingly prevalent. The incidence of postpartum hemorrhage caused by abnormal placental position and growth has increased as well.^[2]^ The 2018 FIGO Guidelines defines placenta accreta spectrum disorders (PASDs) as a group of pathologic disorders presenting with varying degrees of abnormal adherence and penetration of the placental villi into the myometrium. Depending on the depth of placental villous invasion into the uterine wall, PASDs are divided into placenta accreta (grade I), placenta increta (grade II), and placenta percreta (grade III). PASDs have become a major obstetric concern, attracting widespread attention and awaiting a solution.

Pernicious placenta previa (PPP) describes a condition in which the placenta is implanted to the regenerated scars left by a prior cesarean section, which may cause PASD.^[3]^ PPP may lead to hemorrhage at the time of delivery, and may cause emergency peripartum hysterectomy at a rate of 0.24 to 8.7 per 1000 deliveries.^[4]^ Uncontrollable pre-, intra-, and post-operative hemorrhage are primary causes of maternal hysterectomy and neonatal death.^[5–7]^

The pathogenesis of PPP is not fully understood. It is generally believed that PPP is related to primary decidual hypoplasia or a traumatic endometrial defect. To acquire sufficient nutrients, the placenta will extend into even towards the internal cervical orifice, forming a placenta previa. If the placenta extends into the uterine scar, PPP will be resulted.^[5–8]^ The incidence of PPP is increased due to the following factors: short interval between pregnancies; cigarette smoking; use of cocaine; embryo transfer procedures; and a history of placenta previa.^[4,5,7]^ Patients with placental implantation usually have damaged myometrial fibers that interfere with the normal contraction of uterine smooth muscles, thus increasing the risk of postpartum hemorrhage. Given the rich blood supply to the placenta and uterus, placental stripping at delivery will cause a rupture at the implantation site, leading to hemorrhage and a higher risk of death.^[5–7]^ PPP-induced hemorrhage may occur throughout the entire perinatal period. During normal delivery, the placental blood flow rate is 700 to 800 mL/min, but in patients with PPP, the placental blood flow rate may reach up to 1200 mL/min. The collateral blood supply is open in a parturient with placental implantation. Because the placenta is partially stripped during surgery, the blood sinuses at the site of stripping will remain open, which is accompanied by poor uterine contraction. In such cases, the parturient is highly susceptible to fatal hemorrhage. The blood loss can reach up to 3000 to 5000 mL within a short period of time. Depending on the degree of placental implantation, patients with severe placental implantation may have a blood loss > 10,000 mL within a very short time, constituting a pernicious situation. Therefore, PPP prediction and diagnosis in early pregnancy and proper management of PPP at delivery have become major issues in the field of high-risk obstetrics.

The basic principle is to give a comprehensive consideration to blood loss, need for uterine preservation, and prevention of complications.^[3]^ The condition of the parturient can be assessed in pregnancy and before cesarean section, in order to decide on the diagnostic and therapeutic plan, and the measures to prevent and handle prematurity-related and postpartum hemorrhage. Conventional approaches to manage postpartum hemorrhage in PPP include the following: Preventing delivery of the placenta after delivery of the baby; uterine artery ligation if necessary; B-Lynch suture; uterine compression suture; and internal iliac artery ligation. Some specific procedures are also needed locally for the uterus. For example, reinforced sutures, trimming, suture-based hemostasis, and even local excision. A cervical lifting suture and figure of “8” suture can be performed for hemostasis if obvious bleeding spots are identified in the uterine wound.^[9,10]^ The above methods have demonstrated superior hemostatic effects for placental implantation because such patients usually have non-uniform myometrial thickness and poor uterine contractions.^[7]^ Further hemostatic measures will be given after apparent active bleeding stops, such as uterine packing with gauze and uterine massage. Due to extensive pelvic collateral circulation, the blood gushes out uncontrollably in PPP patients and obscures the surgical field. It is almost impossible to expose the site of bleeding and implement hemostatic procedures, which is one reason hysterectomy may be performed indiscriminately in some situations to save the patients’ life.^[9,11]^

Recently, vascular interventional radiation therapy has achieved good efficacy in handling postpartum hemorrhage. Interventional procedures, typically temporary balloon occlusion and vascular embolization, have extensive applications in preventing intraoperative hemorrhage in PPP.^[12]^ Depending on the target site, interventional procedures can be divided into temporary occlusion of the abdominal aorta, bilateral common iliac arteries, and bilateral internal iliac arteries, and bilateral uterine artery embolization. Interventional procedures are usually individualized depending on gestational age, vaginal blood loss, intrauterine fetal survival, and parturient vital signs. The intervention and hemostatic procedures are chosen as appropriate to prevent infections, promote fetal lung maturity, and correct anemia. These procedures are considered important to reduce adverse events, such as intrapartum and postpartum hemorrhage, and performing a hysterectomy.^[13]^ The choice of interventional procedures for the same condition varies across hospitals due to variability in healthcare level and facilities. There are several different interventional procedures for PPP. The commonly used interventional procedures include temporary occlusion of the abdominal aorta, temporary occlusion of the bilateral common (internal) iliac arteries, and bilateral uterine artery embolization, performed alone or in combination with other procedures. Each procedure has its advantages and shortcomings.^[13]^ Below we will introduce the working principle, indications, clinical outcomes, advantages, and disadvantages for each interventional procedure.

This research was a literature review, therefore, the ethical approval for this research was waived by the Institutional Ethic Committee of West China Second University Hospital, Sichuan University.

## 2. Intra-abdominal aortic balloon occlusion

### 2.1. A brief introduction to abdominal aortic balloon occlusion

Intra-aortic balloon occlusion, also known as abdominal aorta clamping (AAC), is a commonly used interventional procedure that has emerged in recent years. This procedure can effectively control intraoperative bleeding and has been proven efficacy in pelvic tumors and fractures.^[14]^ Extravascular and endovascular balloon occlusion are the 2 major approaches for AAC. The former involves making a transabdominal incision, through which the abdominal aorta, about 1 cm long, is dissociated anterior to the vertebral column. The abdominal aorta is encircled with a plasma drainage tube, which is tightened with a vessel clamp until no pulsation is palpable to the common iliac artery, thus indicating successful occlusion. This approach, however, is traumatic and takes a long time to complete.^[14]^ In international countries, AAC has been used to prevent postpartum hemorrhage, achieving good efficacy, and facilitating uterine preservation for PPP patients.^[15–17]^ Clinically, AAC is mainly used for PPP with placenta increta and percreta, or even placental invasion of adjacent organs that cause placenta accreta spectrum (PAS) when the patient wants to reserve uterine.

**Figure 1.**
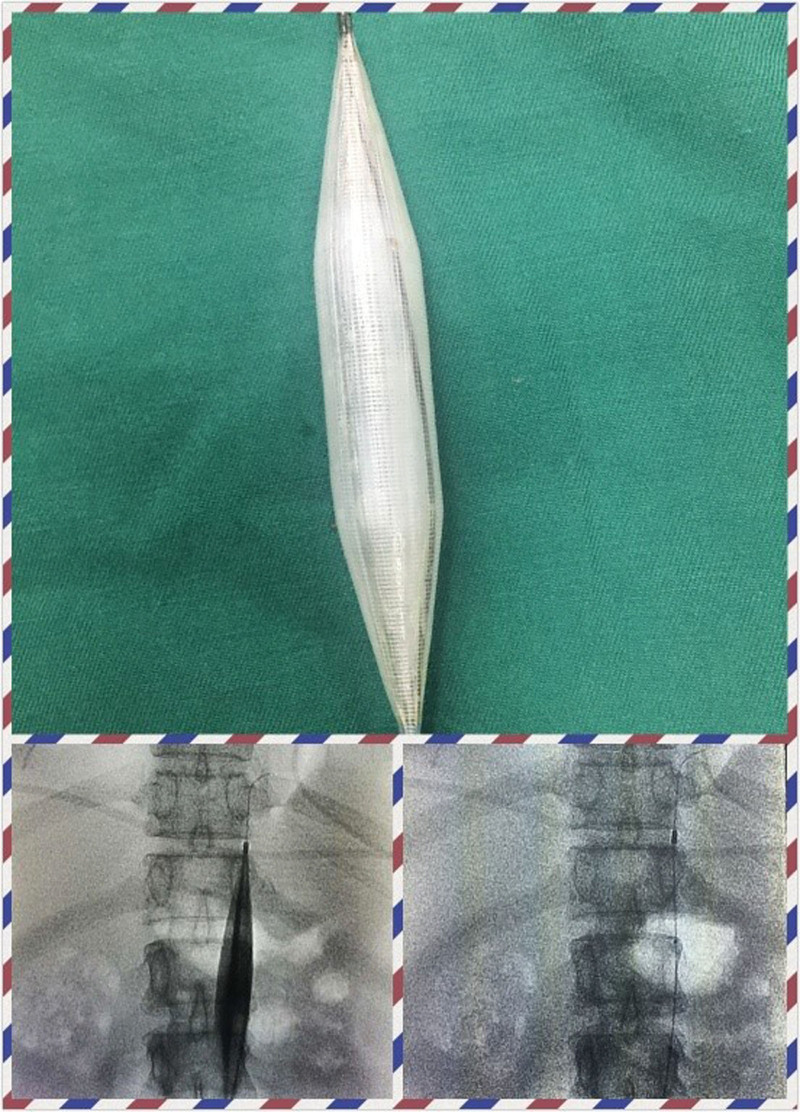
Intraoperative image by the author. The upper image shows pre-dilation of the abdominal aorta artery balloon before occlusion. The lower left image shows the intraoperative balloon occlusion The image in the lower right shows the balloon placement in the renal segment of the abdominal aorta before cesarean section.

The working principle of AAC can be described as follows. The balloon is placed in the aorta. When the balloon expands, the balloon can dramatically reduce the blood supply below the occlusal plane, reducing arterial pressure and sufficiently controlling uterine bleeding in the surgical area. Therefore, the surgeon will have a clean surgical field of view and can conveniently perform venous and arterial suture-based hemostasis at the site of placental stripping. Moreover, this will attenuate coagulation disorders caused by the decrease in coagulation factors due to massive blood volume loss.^[18]^ Before cesarean section, the intra-aortic balloon is placed under X-ray guidance (Fig. 1) and initially remains deflated (Fig. 1). At the time the baby is delivered, normal saline is injected to inflate the balloon, thus to block the blood flow. The balloon inflation can maximally reduce hemorrhage before delivery of the baby, thus reducing placental blood supply and closing the uterine spiral artery. The resulting placental ischemia will make the stripping easy and significantly decrease the blood loss and blood flow at stripping. Placental ischemia can also assist with removal of placental tissues visible to the naked eye.^[19]^ As the placental blood supply decreases, there will be more time to perform suture-based hemostasis and create a clear surgical field of view, which is conducive to adequate hemostasis.^[16,18,19]^

### 2.2. Clinical outcomes and advantages of AAC

Tokue et al^[20]^ report that AAC considerably reduces intraoperative blood loss for PPP patients, thereby reducing the hysterectomy rate during cesarean section.^[21]^ Compared with conventional cesarean section alone, cesarean section plus AAC more effectively reduced surgical blood loss and the hysterectomy rate in PPP patients.^[22,23]^

**Figure 2.**
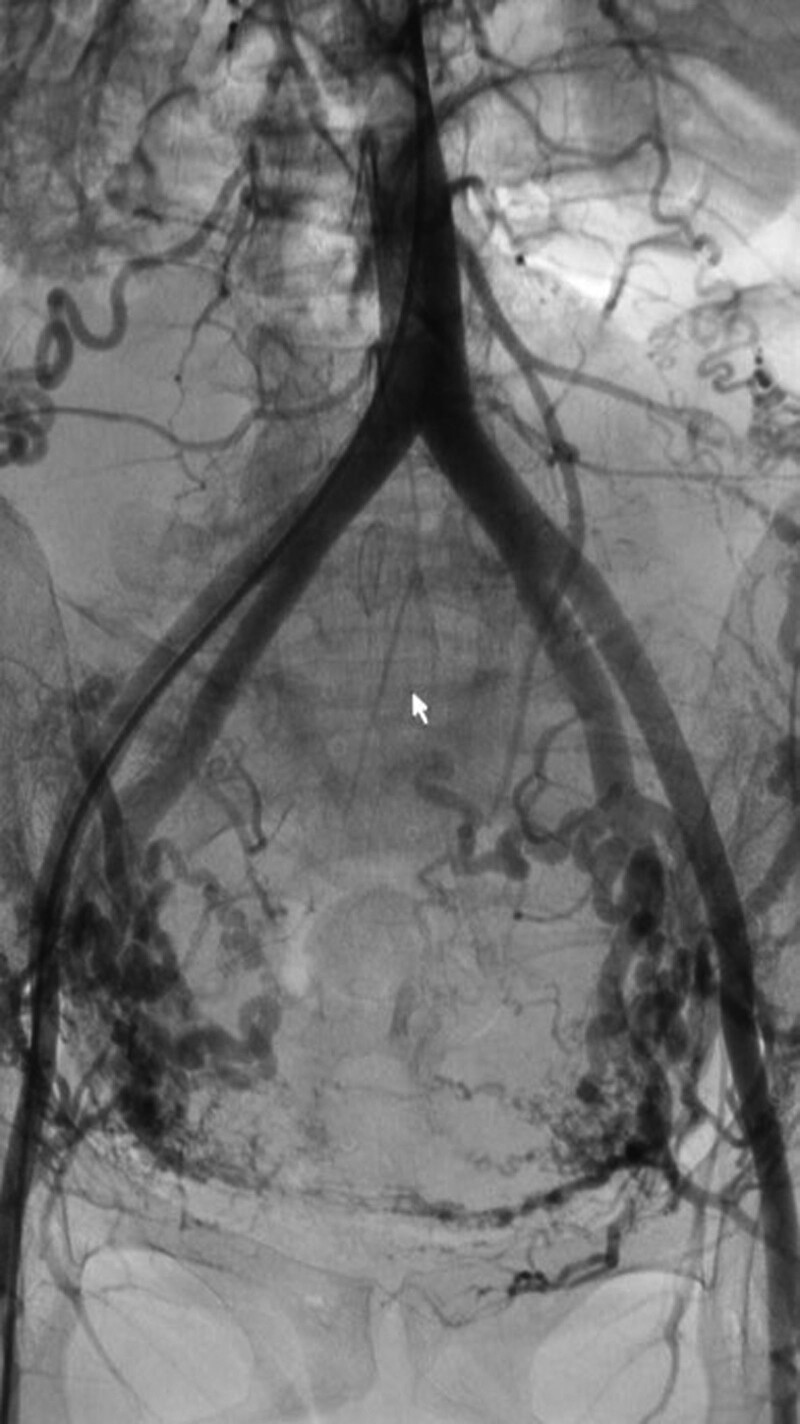
The author performed abdominal aortography in a patient with postpartum hemorrhage. The ovarian artery communicated with the uterine artery. The uterine artery communicated with the superior vesical artery, internal pudendal artery, and branch of the external iliac artery.

The abdominal aorta begins from the aortic hiatus in the diaphragm and travels inferiorly to end at the bifurcation of the bilateral common iliac arteries (the fourth lumbar vertebra). With an average length of 134.90 ± 12.90 mm, the abdominal aorta can be divided into 3 sections. The upper section extends from the 12th thoracic vertebra (T12) to a subdiaphragmatic location. The middle section extends from T12 to the renal artery. The lower section extends from the plane of the renal artery to the bifurcation of the common iliac artery.^[24]^ By placing the balloon between T12 and the diaphragmatic plane, we blocked 61% of the total blood volume. By placing the balloon to the middle position, we blocked 37% of the total blood volume. By placing the balloon to the low position, we blocked approximately 21% of the total blood volume.^[25]^ During cesarean section for PPP patients, intraoperative hemorrhage decreases dramatically after balloon occlusion at a low position; the average blood loss is 1038 mL.^[26]^ The uterus receives blood supply from several arteries, including the ovarian artery, which arises from the abdominal aorta, the superior vesical artery, and the external iliac artery. Abdominal aortic balloon placement at a low position cannot block the blood supply from the ovarian artery. Some researchers suggest placing the abdominal aortic balloon to the middle and high positions to occlude the collateral circulation of the ovarian artery. This maneuver will further reduce the blood loss from the site of placental stripping, and hence intraoperative hemorrhage in PPP patients. The intraoperative blood loss during cesarean section is 586 ± 355 mL using this approach.^[27]^ Abdominal aortic occlusion in a higher position is more conducive to hemostasis, but may adversely influence renal and ovarian functions. In contrast, abdominal aortic occlusion in a lower position does not effectively block collateral vessels supplying the uterus or the blood supply from the ovarian arteries. Therefore, the latter usually fails to achieve the desire hemostatic effect.^[25–27]^ It has been reported that the female body height is linearly related to the length from the sub-renal abdominal aorta to the plane where the mid-point of the right inguinal ligament is situated. This fact can facilitate the rapid and accurate localization of the target plane when placing the balloon.^[28]^ Apart from the uterine artery that supplies blood to the placenta, the superior vesicle artery, internal pudendal artery, and the branch of the external iliac artery are also involved (Fig. 2). The abdominal aorta serves as a sluice gate when occluding the plane of the renal artery, thereby offering a clear surgical field of view.

### 2.3. Clinical controversies

The timing of AAC is still a matter of debate.^[16,17]^ The duration of occlusion depends on the tolerance of organs and tissues for warm ischemia. In PPP, Li et al^[27]^ report balloon dilation once every 5 minutes for AAC in the middle position during obstetric surgery. Another study shows that the time of a single AAC in low position varies somewhere between 15 and 60 minutes for those needing repeated occlusions.^[29]^ Prolonged pelvic surgery may cause prolonged ischemia of tissues and organs, leading to a large number of free radicals, irreversible damage to the organs, and possibly metabolic acidosis in the internal environment.^[27,29]^ AAC should be done in several, repeated attempts to prevent the above situations.

Abdominal aortic balloon placement requires a high level of surgical skill. The blood flow occlusion effect varies across the occlusal planes. Occlusion at a higher plane will block the blood supply to the kidneys.^[30,31]^ The advantages and disadvantages of different occlusion planes have been analyzed above. In case of emergency during AAC, hysterectomy may be necessary, though some difficulty exists and the surgeons require a high skill level. The balloon should be accurately placed, and neither too high nor too low.^[32–34]^

Currently, there has been no unified specification on the use of AAC to treat postpartum hemorrhage. More effort is needed in researching and summarizing the trade-off between the hemostatic effect, positioning the occlusal plane, and reducing prolonged occlusion that may otherwise increase the complications.

## 3. Prophylactic balloon occlusion of bilateral internal or common iliac arteries

### 3.1. A brief introduction to prophylactic balloon occlusion of the internal or common iliac artery

**Figure 3.**
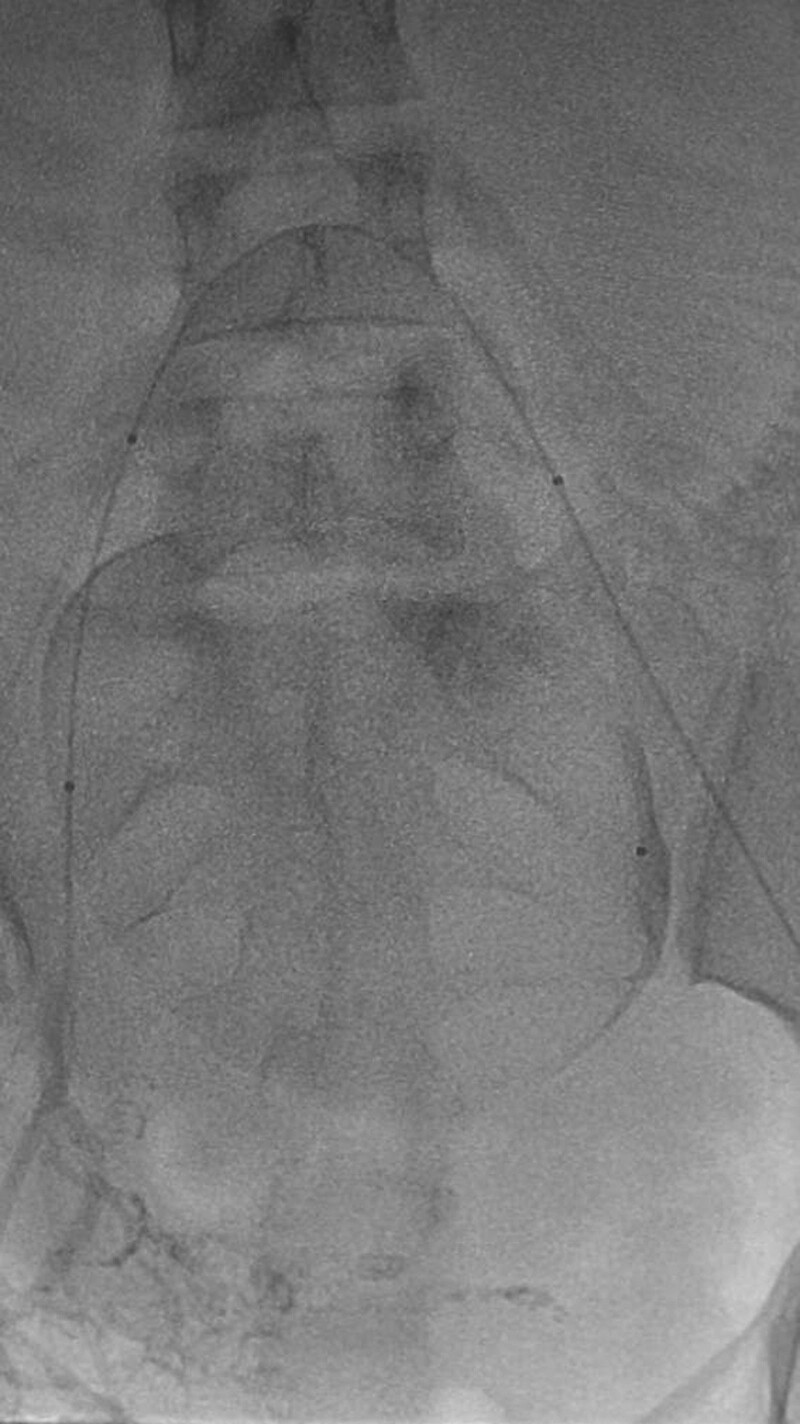
The author placed balloons in the bilateral internal iliac arteries via puncture and catheterization of the bilateral femoral arteries before cesarean section (with bilateral mark points observed from the figure).

**Figure 4.**
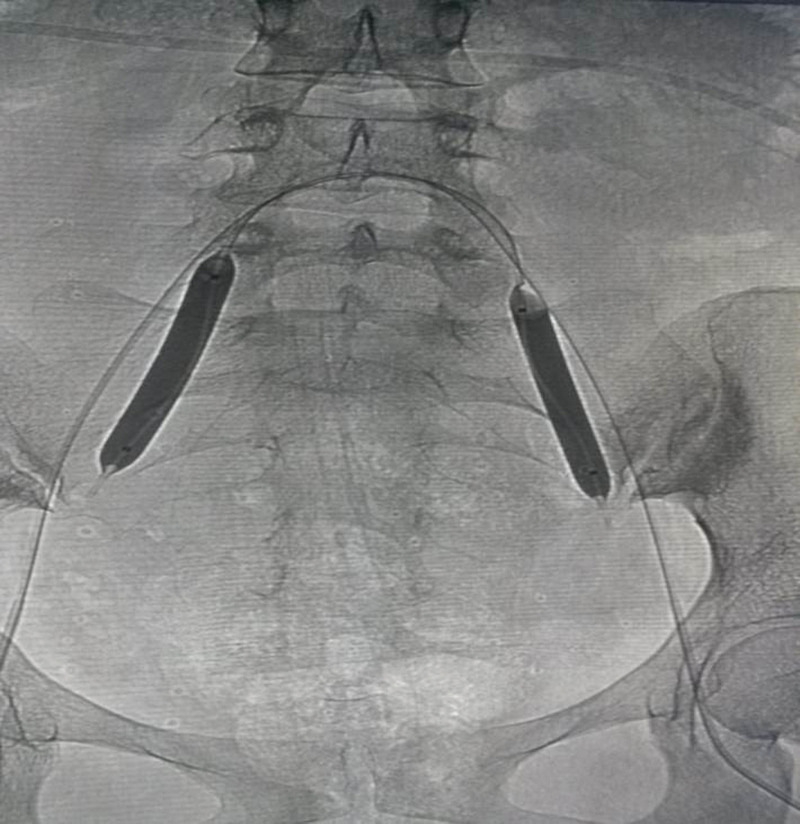
A PPP patient received prophylactic balloon occlusion of the bilateral internal iliac artery before the cesarean section. Bilateral prophylactic balloon occlusion was observed during surgery. PPP = pernicious placenta previa.

In addition to AAC, internal iliac arteries are a commonly used target plan for prophylactic balloon occlusion (PBO). This procedure has been shown to have a good performance in patients with PPP accompanied by PAS.^[35]^ PBO of the bilateral internal iliac arteries blocks the major portion of the uterine blood supply, leaving more time for the subsequent surgery. Specifically, the balloon catheter is first inserted into the internal iliac artery before surgery. The balloon is dilated as appropriate to reduce bleeding and hence the occurrence of fatal hemorrhage without affecting the blood supply to the bilateral lower limbs. This approach has a lesser impact on the circulatory system, and there is little limit on the occlusion time. The occlusion effect, however, is influenced by the degree of placental implantation. This procedure has proven efficacy in PPP complicated by placental implantation.^[36]^ PBO of the internal iliac arteries is performed before cesarean section in PPP patients with placental implantation.^[36]^ After the baby is delivered, the balloon in the internal iliac artery is dilated (Figs. 3 and 4). It is observed during the surgery that the pelvic blood flow was effectively blocked by the balloon. In addition, PBO of common iliac arteries has been widely applied to treat PPP. It has been reported that balloon placement in the common iliac arteries is easier and takes less time. Both the fetus and the mother are exposed to a much lower irradiation dose and for a shorter time.^[37]^

### 3.2. Clinical outcomes and advantages of prophylactic balloon occlusion of the bilateral internal or common iliac arteries

PBO of the bilateral internal or common iliac arteries effectively blocks the pelvic blood flow, thus avoiding massive hemorrhage due to placental stripping. Balloon dilation in this procedure is executed away from the placenta to block pelvic blood flow, thus offering a clear surgical field of view.^[38]^ This procedure can spare more time for obstetricians to achieve complete hemostasis, thereby reducing blood loss and red blood cell transfusion. PBO of the bilateral internal or common iliac arteries also helps reduce the need for hysterectomy and adverse events, such as neonatal asphyxia.^[38]^ According to some reports, PBO of the internal iliac arteries has high importance for reducing blood loss in placental implantation. Because approximately 90% of the uterine blood supply is derived from the anterior branch of the internal iliac artery, balloon occlusion of the bilateral internal iliac arteries effectively blocks the arterial blood flow.^[39]^ Reducing arterial pressure is conducive to coagulation at the wound surface, a clinically significant effect that gives additional time for surgery.^[40]^

Another study shows^[41]^ that balloon occlusion of the internal iliac arteries during cesarean section for PPP patients is not significantly different from a conventional cesarean section for uncomplicated parturients with respect to the hysterectomy rate, neonatal asphyxia rate, surgical time, intraoperative blood loss, and postoperative recovery. The former offers the extra benefits of reducing blood loss and neonatal asphyxia. Nankali et al^[42]^ show that PBO of the internal iliac arteries effectively blocks uterine artery blood flow and reduces blood loss caused by placental stripping. The embolized blood vessels can be recanalized by local suture-based hemostasis of the retained placenta with the assistance from the departments of Interventional Radiology and Vascular Surgery. Uterine preservation is achieved after uterine artery recanalization, thereby reducing various complications, including uterine hemorrhage caused by poor contraction in the lower uterine segment, retained placenta-induced infection, and postpartum hemorrhage.^[42]^ PBO of the bilateral internal iliac arteries reduces intraoperative blood loss by compressing the bilateral uterine arteries, which further lowers the overall surgical risk. In addition, surgeons can decide whether to dilate the balloon or not depending on the intraoperative blood loss and hemostatic effect. This procedure is easy to perform and offers several benefits, including reducing the intraoperative blood loss and preventing ischemic injury or even necrosis of distal tissues caused by prolonged balloon compression.^[43]^ It has been reported that PBO of the internal iliac arteries precludes direct disturbance to the ovarian surface and hence has a lesser impact on ovarian appendages and blood supply.^[44]^ Therefore, PBO of the bilateral internal iliac arteries is feasible and safe for PPP treatment. If there is an ectopic blood supply in the uterus (e.g., ovarian arteries and/or branches of the external iliac arteries supplying the blood), the hemostatic effect by blocking the blood flow in the bilateral uterine arteries or bilateral internal iliac arteries alone will be inferior to blocking the bilateral common iliac arteries or abdominal aorta, at least in theory.^[45]^

### 3.3. Clinical controversies

Hishikawa et al^[46]^ report an initially poor hemostasis in 1 patient with placental implantation, who first received PBO of the internal iliac arteries, with intraoperative balloon filling to block the blood flow in the internal iliac arteries. To address the massive blood loss, Hishikawa et al^[46]^ re-inserted the balloon to the common iliac artery so that the occlusal plane was transferred from the internal iliac artery to the common iliac artery, which dramatically reduced the blood loss. If the bilateral common iliac arteries are occluded by the balloon, the blood supply to the lower limbs is also blocked, apart from blocking the major portion of blood supply to the pelvis. In that case, the risk of distal thrombosis and insufficient blood supply to the lower limbs also increases. Given this fact, we need to strictly control the occlusion time.

**Figure 5.**
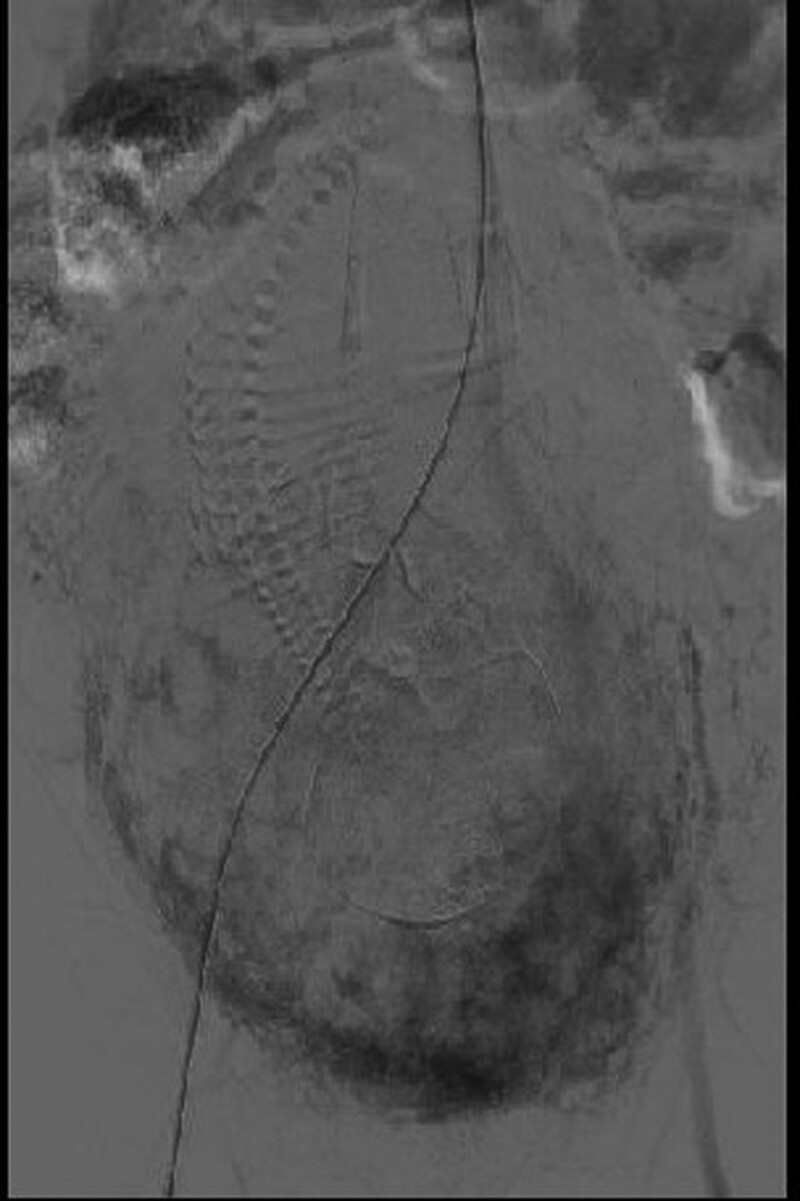
Intraoperative abdominal aortography revealed communication between the uterine artery and the placental sinus in a PPP patient (who received labor induction due to placental implantation). PPP = pernicious placenta previa.

**Figure 6.**
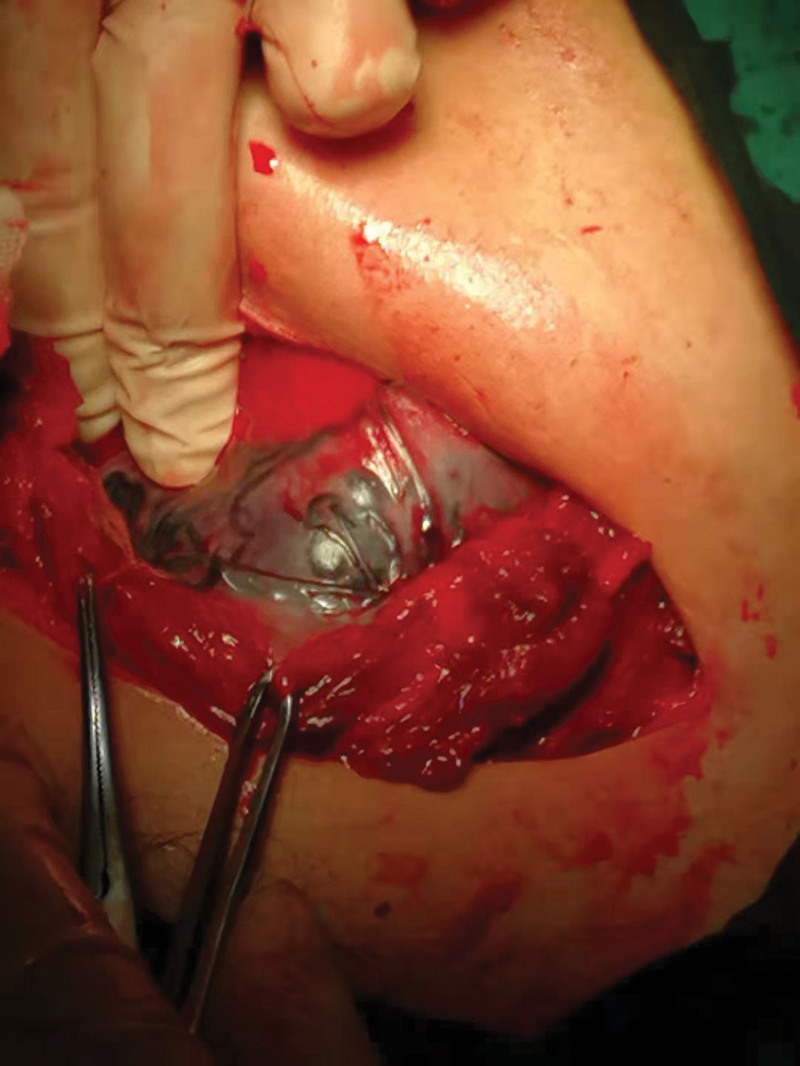
A PPP patient with grade III PAS complicated by a fetal anomaly received labor induction in mid-pregnancy. Bilateral uterine artery embolization was performed before the baby was delivered during cesarean section. It was observed during surgery that the placenta in the lower uterine segment penetrated to the sub-serosal region and had a rich blood supply. PAS = placenta accreta spectrum, PPP = pernicious placenta previa.

Ligation of the internal iliac arteries is traumatic and places a high demand on the skills of the surgeon. The surgical failure rate reaches > 50%.^[47]^ Hysterectomy, which may be required to achieve hemostasis, is at the expense of the patient’s organs and fertility. In addition, 50% to 70% of the ovarian blood supply comes from the ovarian branch. Hysterectomy is very likely to affect ovarian function and cause a series of psychological and physiologic changes, exerting an adverse impact on the quality of life. Occluding the common iliac arteries appears to be more effective and safer than occluding the internal iliac arteries.^[48]^ Occluding the common iliac arteries, however, also carries the risks of ischemia-reperfusion injury to the bilateral lower limbs and kidneys.^[49]^ The time for balloon occlusion of the common iliac arteries is limited, so the duration of blood flow occlusion is short, and the damage to the femoral artery puncture site is large, which deserves due attention from surgeons. Temporary occlusion of the common iliac arteries will also block the blood supply to the lower limbs. A strict control of the occlusion time is necessary. The time of single occlusion is preferably controlled within 40 to 60 minutes, and the shortest time interval between 2 occlusions is approximately 10 minutes.^[50]^ One study suggests that balloon occlusion of the internal iliac arteries does not effectively arrest postpartum hemorrhage due to collateral circulation and reverse flow in uterine venules.^[51]^ Indeed, balloon occlusion of the internal and external iliac arteries has a nominal effect if the collateral circulation is open in patients with grade III PAS. In contrast, blood loss is accelerated due to massive intraoperative hemorrhage from the uterus and opening of blood sinuses if the uterine artery blood flow is not blocked by the conventional rubber tube (Figs. 5 and 6).

Therefore, the feasibility of PBO of the bilateral internal and common iliac arteries to treat PPP depends on the clinical situation, facilities available, and the surgeon’s technical skills. An appropriate choice of the interventional procedure can achieve the desired hemostatic effect and reduce complications.

## 4. Uterine artery embolization

### 4.1. A brief introduction to uterine artery embolization

In recent years, digital subtraction angiography (DSA) – guided bilateral uterine artery embolization (UAE) has been applied to PPP with placenta percreta. PPP is usually combined with severe placental implantation, and the associated massive hemorrhage usually occurs at the moment of placental stripping or near fetal delivery. UAE can effectively control postpartum hemorrhage.^[52]^

UAE involves DSA of the internal iliac artery to determine the site of bleeding. Then, the embolizing agent is injected into the uterine artery to block the arterial lumen and reduce uterine arterial pressure and blood flow, which is conducive to embolization. As the uterine blood supply decreases, the uterine smooth muscles are in an ischemic state, which promotes uterine smooth muscle contraction and imposes compression on the blood sinuses at the site of placental stripping. As the uterine blood loss decreases, so does the incidence of adverse events.^[53]^ UAE is commonly used to treat uncontrollable postpartum hemorrhage, as preoperative bilateral UAE can cause fetal asphyxia.^[54]^

### 4.2. Clinical outcomes and advantages of uterine artery embolization

UAE blocks the blood supply to the retained placenta that is embedded in the myometrium. As a result, the placenta may degenerate, and become ischemic and necrotic within a short period of time, which helps prevent complications related to a retained placenta, including poor uterine rejuvenation and postpartum hemorrhage. This procedure improves the therapeutic effect.^[55]^ Researchers have found that the blood loss is 1000 to 4000 mL in patients undergoing interventional procedures due to hemorrhage. Although this procedure can reduce the puerperal mortality and hysterectomy rates, the patients may experience shock-induced disseminated intravascular coagulation. UAE should be performed as early as possible during cesarean section. The timing of the interventional procedure should be optimized to avoid fetal exposure to DSA.^[56]^ The DSA-guided UAE can correctly determine the site of bleeding, preventing uterine hemorrhage and reducing the hysterectomy rate. This procedure is also conducive to future pregnancy and lowering the hysterectomy rate.^[57,58]^ The embolizing agent used in the procedure is a soluble substance, which can be absorbed within 2 weeks postoperatively. Pelvic organs are unique because after a bilateral UAE, the uterine body will be supplied by collateral circulation. Therefore, ischemic-induced necrosis of the uterine body can be prevented, which is conducive to future pregnancy. In addition, bilateral UAE can block the placental blood supply, resulting in ischemia and apoptosis of trophoblast cells.^[59]^

To conclude, bilateral UAE temporarily blocks uterine blood circulation and establishes anastomoses between the visceral branches of the pelvic vessels and between the visceral and parietal branches of the pelvic vessels to provide sufficient blood supply to the uterus. Moreover, this procedure is minimally invasive and safe and capable of achieving fast hemostasis and uterine preservation. UAE has been reported to achieve a higher success rate in hemorrhage arrest.^[53]^

### 4.3. Clinical controversies

**Figure 7.**
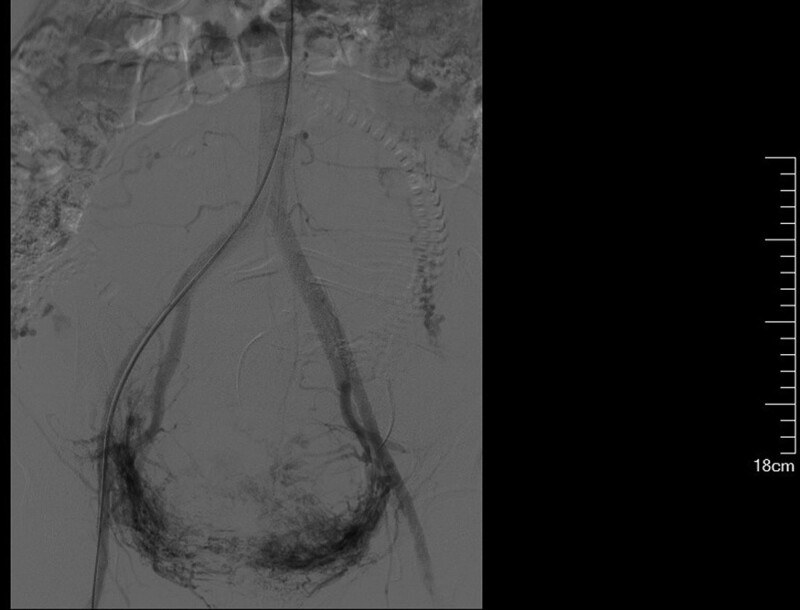
Preoperative abdominal aortography was performed for a patient scheduled for uterine artery embolization due to postpartum hemorrhage. The tubal branch of the left ovarian artery was opened to the uterine fundus. The uterus passed through the main sacral ligament vessels of the descending branch of the uterine artery to the contralateral descending branch of the uterine artery.

One study has pointed out that the effective rate of uterine artery ligation is only 50% and this procedure is associated with a high incidence of complications,^[47]^ probably due to the rich collateral circulation in the uterus and the pelvic floor. UAE is an auxiliary hemostatic procedure following a cesarean section with AAC. For PPP, it has been shown that UAE of the only arteries supplying the uterus has an excellent hemostatic effect for general postpartum hemorrhage; however, the efficacy is limited for hemorrhage at the site of placental stripping due to PPP with placental implantation. The uterine blood supply and collateral circulation are rich in pregnancy. Apart from the uterine arteries, other supplying vessels include the internal iliac arteries, venous plexuses, branches of ovarian arteries, and abnormally proliferative vessels. Embolizing the uterine arteries alone has limited efficacy. Some parturients may still have refractory bleeding and require a hysterectomy due to difficulty in stripping the implanted placenta, individual factors, and coagulopathy.^[60]^ Suture ligation to block local venous hemorrhage is equally important because venous sinuses and uterine spiral arteries are also involved in uterine hemorrhage. Hemorrhage in PPP is mainly contributed by the descending branches of the uterine arteries and some ascending branches of the uterine arteries [personal observation]. Therefore, embolizing the descending branches of the uterine arteries is also important, otherwise, the ovarian collateral vessels will be open and supply blood to the contralateral side via the ovarian fundus and body or even the cardinal ligament (Fig. 7). Internal iliac artery embolization is not recommended if the culprit vessels of the uterine arteries can be precisely embolized. Internal iliac artery embolization may lead to reflux of the embolization particles into the external iliac artery and gluteal arteries. Excessive embolism may result in atrophy and pain of the ischemic muscles. Open ovarian arteries can also reach the ovarian fundus (Fig. 7), rather than descending to the lower uterine segment or the cervix.

## 5. Discussion

### 5.1. Comparisons of different methods

Wei et al^[61]^ conclude that for PPP with placenta percreta, AAC outperforms balloon occlusion of the bilateral internal iliac arteries with respect to intraoperative and 24-h blood loss. As discussed above, AAC is associated with a shorter average surgical time and shorter balloon occlusion time, lower rate of UAE, and small fetal radiation dose than balloon occlusion of the internal iliac arteries.^[20,44]^ Wang et al^[62]^ also report that AAC outperforms balloon occlusion of internal iliac arteries. Another cohort study indicates that balloon occlusion of common iliac arteries and the sub-renal aorta achieves a better hemostatic effect and better perioperative outcomes than internal iliac arteries for patients with placenta increta.^[63]^ Zhou et al^[38]^ perform a retrospective study of patients with placenta increta undergoing surgery. AAC outperforms UAE in blood loss, amount of blood transfusion, duration of radiotherapy, radiotherapy dose, hysterectomy rate, surgical time, length of stay in the intensive care unit, and incidence of complications.^[38]^ Tan et al^[64]^ believe that compared with balloon occlusion of the internal iliac arteries in patients with PPP and an adherent placenta, AAC only reduces the occlusion time, radiation dose, and surgical time. Yu et al^[11]^ are of the opinion that for PPP with placenta percreta, AAC outperforms balloon occlusion of the bilateral internal iliac arteries with respect to intraoperative and 24-hours blood loss (*P* < .05); however, such differences are of no statistical significance for placenta accreta or placenta increta. The probability of an Apgar score (11 minutes) < 8 is significantly higher in the AAC group than the group receiving balloon occlusion of the internal iliac arteries (*P* < .05). AAC can result in a clearer surgical field of view, sparing more time for surgeons to perform fine suture of the bleeding points. Balloon occlusion of the internal iliac arteries involves continuous arterial occlusion, while AAC involves an alternation between arterial occlusion and opening. The latter procedure allows for a more comprehensive search for bleeding points. The reasons for the difference in Apgar scores between the 2 groups were as follows. Balloon occlusion of the internal iliac arteries blocks less pelvic blood flow than AAC, and therefore has a lower probability of ischemic damage. According to another study, AAC more effectively reduces blood loss, surgical time, and fetal radiation dose than balloon occlusion of the internal iliac arteries in patients undergoing an emergency cesarean hysterectomy.^[65]^ The choice of specific interventional procedures is open to discussion, given the diversity and complexity of clinical situations. According to a meta-analysis, balloon occlusion of the internal iliac arteries and AAC are most commonly practiced. In addition, the latter is associated with less blood loss and a lower hysterectomy rate.^[66]^

UAE can effectively reduce intraoperative blood loss and the need for blood transfusion in PPP patients after cesarean section and AAC, thereby reducing the risk for a hysterectomy.^[17,42]^ In addition, the rate of UAE, the rate of abdominal aorta ligation, and the rate of intrauterine packing with iodophor gauze are significantly lower in the former group than the latter group (*P* < .05). The 2 procedures differ in the rate of UAE because the abdominopelvic collaterals are open, continuously supplying the uterine arteries. When the abdominal aorta is blocked, the blood supply from the collaterals to the pelvic cavity decreases significantly.

According to one study based on angiography, the round ligament artery supplying the uterine collaterals might be a risk factor for hemorrhage in PPP during cesarean section.^[67]^ Considering the structural complexity in PPP, many researchers have recommended the combined use of interventional procedures as an optimized scheme for individual patients. Liu et al^[68]^ report that among 31 patients receiving AAC, 9 have hemorrhage after the balloon is released. These patients then underwent embolization of the uterine or ovarian arteries, which effectively stopped the postpartum hemorrhage.^[68]^ D’Souza et al^[69]^ also show that balloon occlusion of the bilateral internal iliac arteries plus UAE is an effective method to control intraoperative blood loss and preserve the uterus during invasive cesarean section in PPP. The success rate of uterine preservation reaches up to 70%.^[69]^ Soyer et al^[70]^ find that the hysterectomy rate is 15.5% in patients receiving UAE versus 76.5% in those receiving balloon occlusion of the internal iliac arteries. Thus, UAE may be the preferred choice for fertility preservation.^[70]^ Another study suggests that balloon occlusion with a hypophysin infusion significantly reduces the blood loss, amount of blood transfusion, and hysterectomy rate in PPP patients.^[71]^

### 5.2. Different methods for different grading

**Figure 8.**
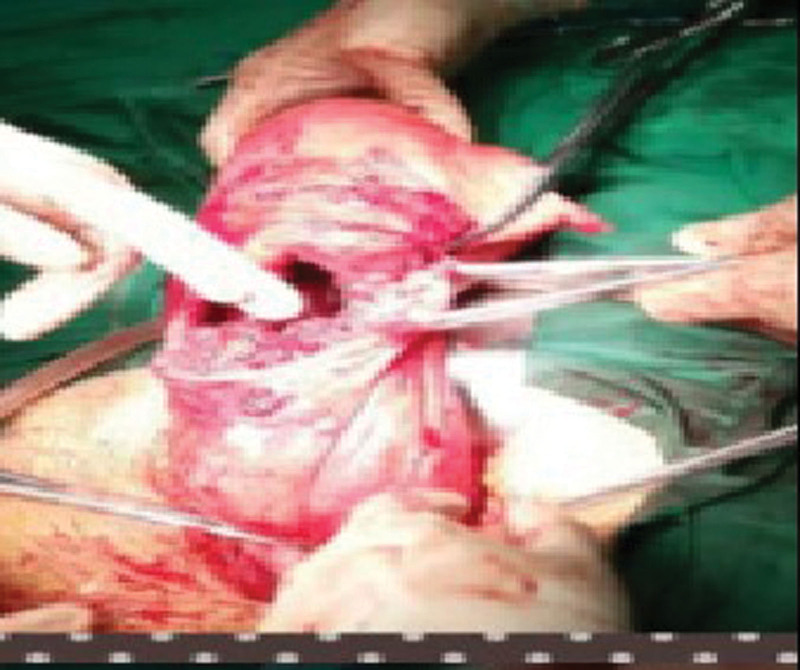
Bilateral uterine artery embolization was performed after cesarean section following AAC in a hybrid operating room for a PPP patient. Hysterectomy was performed due to difficulty in the right uterine repair, and the uterus was sac-like. AAC = abdominal aorta clamping, PPP = pernicious placenta previa.

**Figure 9.**
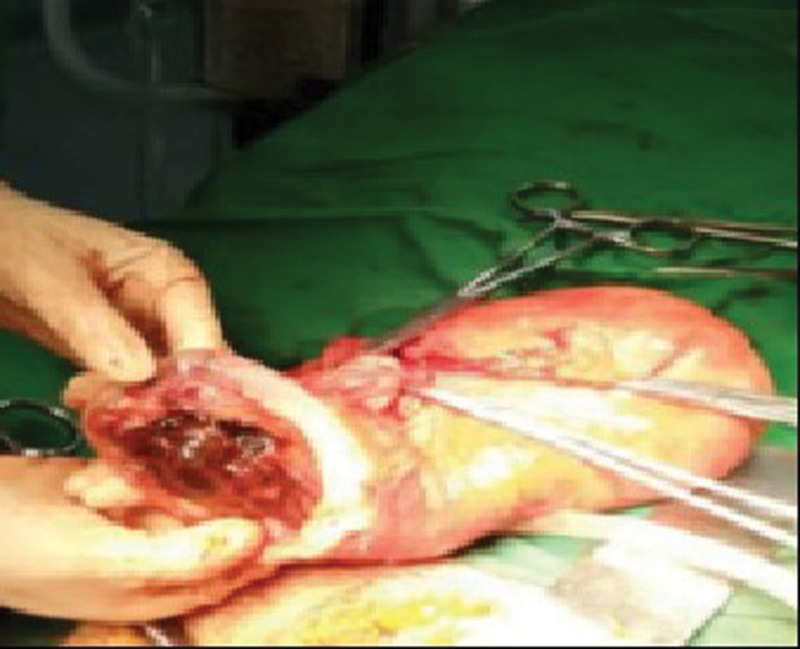
Bilateral uterine artery embolization was performed after cesarean section following AAC in a PPP patient. The uterus was sac-like, and hysterectomy was performed due to poor uterine contraction. AAC = abdominal aorta clamping, PPP = pernicious placenta previa.

**Figure 10.**
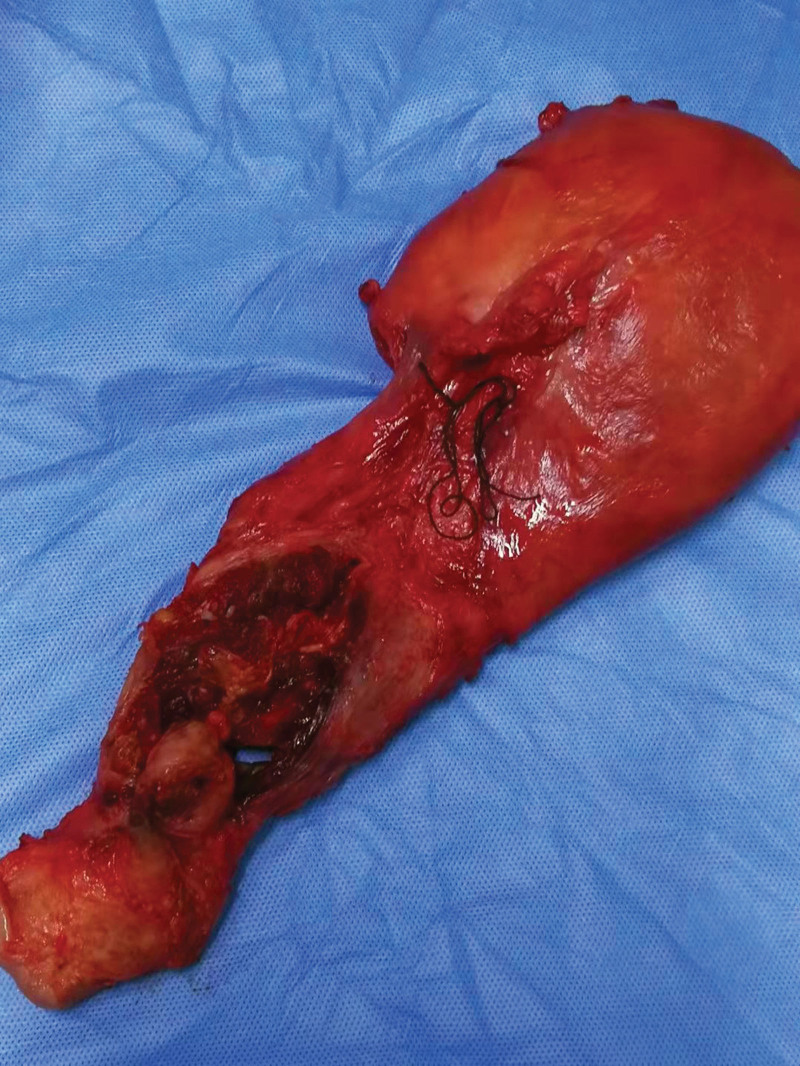
Placental tissues penetrating the bladder were noted during cesarean delivery in a PPP patient with grade III PAS. A total hysterectomy was performed. PAS = placenta accreta spectrum, PPP = pernicious placenta previa.

**Figure 11.**
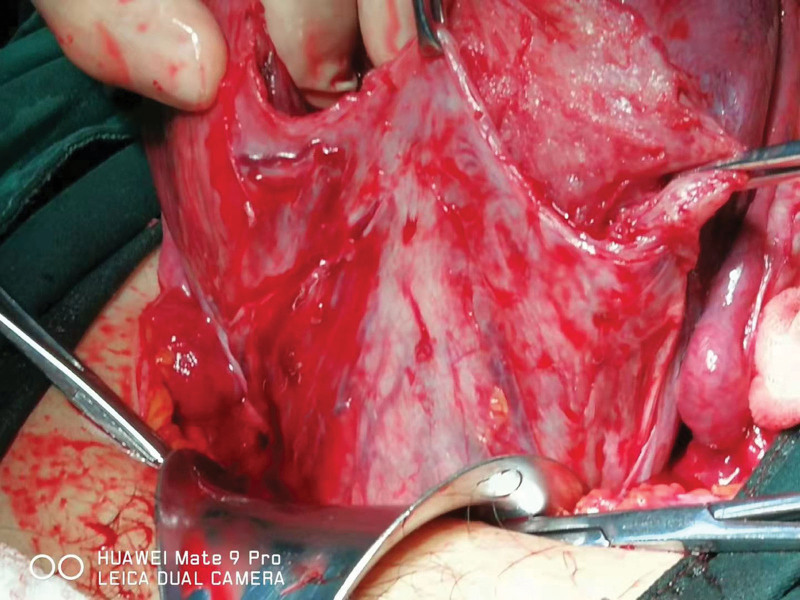
Placental tissues penetrating the bladder were noted during cesarean delivery in a PPP patient with grade III PAS. PAS = placenta accreta spectrum, PPP = pernicious placenta previa.

Based on a literature review and our own clinical experience [personal observation], temporary balloon occlusion of the abdominal aorta is more easily acceptable to interventional surgeons and PPP patients with a rich placental blood supply, especially patients with grade III PAS. Specifically, the balloon is first placed in the abdominal aorta before cesarean section. Then, the need for UAE is assessed postoperatively based on blood loss and the preoperative hemostatic effect. Repair or hysterectomy may be performed following UAE without prophylactic balloon placement for grade I-II PAS patients if postpartum hemorrhage occurs (Figs. 8–11) to achieve dual hemostatic effects.^[52]^ Prophylactic balloon placement in internal iliac arteries may be satisfactory for patients with grade I-II PAS, in which collateral arteries are not opened and the placenta is only supplied by uterine arteries. UAE is rarely needed as a subsequent complementary treatment. In addition, findings from recent studies may facilitate the management of postpartum hemorrhage by balloon occlusion plus medications.

Common adverse reactions associated with interventional procedures include the following: fetal X-ray exposure; local pain caused by embolization-induced tissue ischemia; fevers; lower extremity thrombosis; intimal injury of blood vessels; ischemia-reperfusion injury; arterial tears; and endometrial peeling.^[11]^ Surgeons should be more aware of the importance of preoperative prophylaxis, which functions synergistically with interventional procedures and postoperative management to reduce complications and safeguard fetal and maternal safety.

## 6. Conclusion

For these PPP patients accompanied by PAS, early B-mode ultrasound and magnetic resonance imaging are necessary to confirm the diagnosis and determine the location and anatomy of the uterus-supplying blood vessels for a more precise preoperative disease evaluation. The surgical approach and auxiliary interventional procedure should be chosen appropriately based on the hospital infrastructure and technical level. Several treatments may be delivered in combination if necessary to achieve the desired hemostatic effects.

In patients with grade III PAS with extensive sub-serosal penetration of the uterus and repair difficulty, repair or hysterectomy may be performed following UAE if there is a hybrid operating room. For grade I-II PAS (shallow placental implantation), prophylactic balloon occlusion may not be necessary before surgery. UAE can be performed in cases with postoperative hemorrhage.

## Author contributions

**Conceptualization:** Hu Zhao.

**Data curation:** Hu Zhao, Mou Han, Xue Xiao.

**Formal analysis:** Hu Zhao, Qiong Wang, Mou Han, Xue Xiao.

**Investigation:** Qiong Wang, Mou Han, Xue Xiao.

**Methodology:** Qiong Wang, Xue Xiao.

**Supervision:** Hu Zhao.

**Validation:** Qiong Wang, Mou Han.

**Visualization:** Mou Han.

**Writing – original draft:** Xue Xiao.

**Writing – review & editing:** Xue Xiao.
